# Machine learning model for ACLF risk in decompensated cirrhosis

**DOI:** 10.3389/fmed.2026.1948234

**Published:** 2026-10-07

**Authors:** Liwen Chen, Peipei Ren, Yi Kang

**Affiliations:** Department of Infectious Diseases, Henan Provincial People’s Hospital, Zhengzhou University People’s Hospital, Zhengzhou, Henan, China

**Keywords:** acute-on-chronic liver failure, decompensated cirrhosis, machine learning, random forest, SHAP, nomogram, prediction model, lactate

## Abstract

**Objective:**

To develop a machine learning–based predictive model for acute-on-chronic liver failure (ACLF) risk in patients with decompensated cirrhosis using baseline clinical indicators at hospital admission.

**Materials and methods:**

This retrospective cohort included 360 decompensated cirrhosis patients with intensive monitoring needs or infection/circulatory risk factors, randomly divided into training and validation sets at a 7:3 ratio. Three models—random forest, support vector machine, and logistic regression—were constructed. Model performance was evaluated using the area under the receiver operating characteristic curve (AUC), calibration curves, and decision curve analysis (DCA). SHapley Additive exPlanations (SHAP) were employed for model interpretability.

**Results:**

In the training set, 52 patients (20.6%) experienced ACLF. Multivariate analysis identified total bilirubin (TBil) (OR = 1.051, 95% CI: 1.030–1.073, *P* < 0.001), arterial lactate (OR = 1.341, 95% CI: 1.058–1.700, *P* = 0.015), and white blood cell count (WBC) (OR = 1.111, 95% CI: 1.007–1.227, *P* = 0.036) as independent risk factors, whereas prothrombin time activity (PTA) (OR = 0.961, 95% CI: 0.941–0.982, *P* < 0.001) was protective. The random forest model showed comparable performance to logistic regression with training/validation AUCs of 0.833 (95% CI: 0.757–0.910) and 0.700 (95% CI: 0.555–0.845). All models showed acceptable discrimination and net clinical benefit on DCA. Calibration was good in the training set but moderate in the validation set (calibration slopes 0.580–0.620). SHAP importance ranking placed PTA, TBil, WBC, and lactate as the top predictors.

**Conclusion:**

The random forest model showed acceptable training performance, but the wide validation AUC confidence interval suggests limited stability, likely due to small sample size and few events. External validation is needed before clinical use. Nonetheless, the model shows potential for early warning and risk stratification in decompensated cirrhosis.

## Introduction

Acute-on-chronic liver failure (ACLF) is characterized by acute deterioration of liver function in patients with pre-existing chronic liver disease, presenting as a complex clinical syndrome marked by multi-organ failure, systemic inflammatory response, and high short-term mortality. Acute decompensation of cirrhosis constitutes the predominant context for ACLF development, with approximately 31% of hospitalized patients with cirrhosis progressing to ACLF due to acute complications. A global meta-analysis revealed that the incidence of ACLF in patients with decompensated cirrhosis reaches 35%, with the highest rate of up to 65% in South Asia; the 28-day mortality rate is approximately 38.6%, increasing to 45.8% at 90 days (1, 2). The European CANONIC cohort reported that, according to EASL-CLIF diagnostic criteria, the prevalence of ACLF in patients with cirrhosis was 29.3% (3, 4), with 28-day and 90-day transplant-free survival rates of 58.4% and 32.2%, respectively. ACLF progresses rapidly with an extremely poor prognosis; the short-term mortality rate with comprehensive medical management ranges from 50% to 90%, posing a serious threat to patient survival and imposing a substantial burden on healthcare resources.

The diagnosis and grading of ACLF currently rely primarily on the EASL-CLIF organ failure assessment system, which integrates the functional status of six organ systems—liver (total bilirubin), kidney (creatinine or renal replacement therapy), coagulation (international normalized ratio), circulation (use of vasoactive agents), respiration (oxygenation index), and brain (hepatic encephalopathy grade)—in the context of acute decompensation of cirrhosis. ACLF is graded (grades 1–3) based on the number and combination of organ failures: grade 1 includes single kidney failure, or single liver/coagulation/circulatory/respiratory failure combined with renal or cerebral dysfunction, or single cerebral failure combined with renal dysfunction; grade 2 comprises two organ failures; and grade 3 comprises three or more organ failures (5, 6). However, for hospitalized patients with decompensated cirrhosis who have not yet developed ACLF, there remains a lack of systematic and quantitative early warning tools to identify the short-term risk of progression to ACLF. Clinical assessment of these patients largely relies on single indicators (e.g., a single measurement of total bilirubin or prothrombin time activity) or conventional prognostic scores (e.g., MELD score, Child-Turcotte-Pugh score). Although these approaches provide some reference value, they are insufficient to comprehensively capture the dynamic evolution of multi-system compensation and decompensation preceding ACLF onset (7, 8). Therefore, developing a risk prediction model for new-onset ACLF in patients with decompensated cirrhosis shortly after hospital admission is of great significance for achieving early warning, optimizing tiered management, and improving patient outcomes.

In recent years, machine learning (ML) methods have demonstrated promising applications in prognostic prediction within hepatology, leveraging their advantages in processing high-dimensional, non-linear data and capturing complex interactions among variables. Several studies have successfully constructed ML-based prediction models for short-term prognosis (e.g., 28-day mortality) in patients with ACLF (9). Random forest, support vector machine, and logistic regression are among the most widely used algorithms in clinical prediction modeling. Nevertheless, the “black-box” nature of ML models has limited their clinical acceptance to some extent. SHapley Additive exPlanations (SHAP) values, a game theory-based interpretability method, can quantify the contribution and direction of each input feature to model predictions, effectively addressing this limitation. To date, studies specifically combining ML with SHAP-based interpretability analysis for predicting new-onset ACLF in patients with decompensated cirrhosis after hospital admission remain limited.

Accordingly, this retrospective study collected baseline clinical data from hospitalized patients with decompensated cirrhosis. Independent predictors of ACLF were identified through univariate analysis, collinearity diagnostics, and multivariate logistic regression with forward stepwise selection. Three ML prediction models—random forest, support vector machine, and logistic regression—were constructed and evaluated using receiver operating characteristic (ROC) curves, calibration curves, and decision curve analysis (DCA) to assess discriminative ability, calibration, and clinical net benefit. SHAP values were employed to interpret the random forest model, and a visual nomogram scoring system was ultimately developed. This study aimed to provide a quantitative decision-support tool for early identification of high-risk ACLF patients among those requiring intensive monitoring, facilitating individualized tiered management in this specific population.

## Materials and methods

### Study population

This study was a single-center retrospective cohort study that consecutively enrolled 360 hospitalized patients diagnosed with decompensated cirrhosis at our institution between February 2023 and February 2026. The target population was operationally defined as patients with decompensated cirrhosis who required intensive monitoring (e.g., ICU admission, step-down unit care) or were at high risk of infection or circulatory instability (defined as suspected sepsis, hypotension with systolic blood pressure < 90 mmHg, or respiratory distress requiring supplemental oxygen). Among the 480 initially screened patients, 120 (25.0%) were excluded: 42 had ACLF at baseline, 18 were already receiving vasoactive agents or CRRT at baseline, and 60 did not have arterial lactate measured or did not meet the criteria for intensive monitoring/high-risk status, leaving 360 patients for the final analysis. Clinical data were extracted from the hospital electronic medical record system and the laboratory database. Owing to the retrospective design and anonymization of all data, the institutional ethics committee waived the requirement for informed consent.

The required sample size was estimated using PASS 2021 and the “pwr” package in R, with a significance level of *α* = 0.05 and a power of 1 − *β* = 0.80. Based on our pilot data and published literature, the cumulative incidence of new-onset ACLF within 28 days among hospitalized patients with decompensated cirrhosis was estimated at 15%–20% (10). Using a conservative estimate of 15% and accounting for a 10% data missing rate, the minimum required sample size was calculated as 338. Additionally, in accordance with the Events Per Variable (EPV) principle for prediction model development (at least 10 events per predictor parameter), we planned to include no more than 5 predictor parameters, requiring at least 50 positive events. The study ultimately enrolled 360 patients, with 52 ACLF events in the training set; the final model included four predictors (PTA, TBil, Lac, and WBC), resulting in 4 model parameters with an EPV of 52/4 = 13.0, which satisfied the sample size requirement for modeling.

Inclusion Criteria: (1) Age ≥ 18 years and ≤80 years; (2) met the clinical diagnostic criteria for decompensated cirrhosis, with diagnosis based on typical clinical signs and symptoms, abnormal liver function and coagulation parameters, and imaging findings (ultrasonography, CT, or MRI) confirming cirrhosis with portal hypertension and hypersplenism; (3) completed all baseline clinical and laboratory assessments within 24 h of admission, with critical data missing rate ≤ 10%; (4) clinical data (demographics, etiological data, vital signs, laboratory tests, treatment records) were complete and traceable.

Exclusion Criteria: (1) Met the EASL-CLIF diagnostic criteria for ACLF at admission, defined as the presence of any of the following organ failures: liver failure (total bilirubin ≥ 12 mg/dL, approximately 205 µmol/L), kidney failure [creatinine ≥ 2.0 mg/dL, approximately 177 µmol/L, or already receiving continuous renal replacement therapy (CRRT)], coagulation failure [international normalized ratio (INR) ≥ 2.5], circulatory failure (requiring vasoactive agents to maintain blood pressure), respiratory failure (PaO_2_/FiO_2_ ≤ 200 or requiring invasive mechanical ventilation), or cerebral failure (hepatic encephalopathy grade ≥ 3); (2) concurrent hepatocellular carcinoma or other malignancies; (3) concurrent severe cardiovascular or cerebrovascular disease (e.g., New York Heart Association functional class ≥ III, acute myocardial infarction, acute stroke); (4) concurrent human immunodeficiency virus infection or other severe immunodeficiency disorders; (5) pregnant or lactating women; (6) prior liver transplantation; (7) critical data missing rate > 10% or lost to follow-up; (8) age < 18 years or >80 years.

### Data collection

The following information was collected through the hospital electronic medical record system, laboratory database, and picture archiving and communication system.

General Data: Included age (years), sex (male/female), body mass index (BMI, kg/m^2^), etiology of cirrhosis (chronic hepatitis B, alcoholic liver disease, autoimmune liver disease, other), alcohol consumption history (yes/no), and smoking history (yes/no).

Clinical Complications and Vital Signs: Included hepatic encephalopathy grade (grades 0–2, according to the West Haven criteria 11), ascites (yes/no), body temperature (°C), heart rate (beats/min), and systolic blood pressure (mmHg).

Laboratory Parameters: Fasting peripheral venous blood samples were collected within 24 h of admission and uniformly tested by our institutional laboratory. Parameters included: prothrombin time activity (PTA), total bilirubin (TBil), alanine transaminase (ALT), aspartate transaminase (AST), albumin (ALB), creatinine (Cr), platelet count (PLT), and white blood cell count (WBC). Arterial blood lactate (Lac) was measured within 24 h of admission using radial or femoral arterial blood samples, uniformly tested by our institutional blood gas analyzer. Of note, all patients in this study underwent arterial blood gas analysis during hospitalization due to intensive care admission or the presence of infection/circulatory instability risk; this parameter is not routinely screened in general wards, and the applicability of the model should be carefully considered when extrapolating to broader populations.

Therapeutic Interventions: Recorded whether vasoactive agents were used within 24 h of admission (yes/no, including terlipressin, norepinephrine, dopamine, etc.) and whether CRRT was received (yes/no).

All laboratory tests were performed in our institutional laboratory, with instrument calibration and quality control strictly adhering to standard operating procedures. Clinical data were independently extracted and cross-checked by two clinicians with intermediate or senior professional titles, with discrepancies adjudicated by a third physician with a senior title.

Baseline tests were defined as those performed within 24 h of admission. For patients who received vasoactive agents in the emergency department or within 24 h of admission, these were classified as baseline treatments; if such treatments met the EASL-CLIF criteria for circulatory failure (i.e., use of vasoactive agents to maintain blood pressure), the patients were excluded at baseline.

### Outcome definition and grouping criteria

Outcome Definition: The primary outcome of this study was new-onset ACLF within 28 days of admission. Outcome determination was independently performed by two hepatologists with senior professional titles, strictly following the EASL-CLIF 2013 diagnostic criteria, namely, the new occurrence of any of the following organ failures on the basis of decompensated cirrhosis: liver failure (TBil ≥ 12 mg/dL, approximately 205 *μ*mol/L), kidney failure (Cr ≥ 2.0 mg/dL, approximately 177 *μ*mol/L, or new initiation of CRRT), coagulation failure (INR ≥ 2.5), circulatory failure (new initiation of vasoactive agents to maintain blood pressure), respiratory failure (PaO_2_/FiO_2_ ≤ 200 or new initiation of invasive mechanical ventilation), or cerebral failure (new attainment of hepatic encephalopathy grade ≥ 3). If a patient died within 28 days, the last laboratory and treatment records within 48 h prior to death were reviewed to determine whether the above criteria were met; if the outcome could not be determined due to missing critical data, the case was retained as a non-event (ACLF-negative) in the primary analysis. All outcome determinations were performed blinded to baseline predictors. It should be explicitly clarified that the “new initiation of vasoactive agents” or “new initiation of CRRT” used as outcome criteria in this study specifically refers to treatment escalation events occurring for the first time during hospitalization after completion of baseline data collection within 24 h of admission. Patients already receiving the above treatments at baseline (within 24 h of admission) had been excluded, ensuring a strict temporal separation between treatment initiation in outcome determination and baseline predictor variables, thereby avoiding circular validation issues.

Grouping Criteria: (1) All enrolled patients were randomly divided into a training set (70%, for feature selection, model training, and parameter optimization) and a validation set (30%, for internal validation and performance evaluation) at a 7:3 ratio. (2) Patients in the training set were further categorized into the ACLF group (positive group) and non-ACLF group (negative group) based on whether the composite clinical endpoint occurred within 28 days of admission. All outcome determinations were independently assessed by two hepatologists with senior professional titles, with discrepancies adjudicated by a third senior physician, and the outcome assessment process was blinded to baseline predictors.

For patients discharged before day 28, active follow-up was performed via telephone contact at day 28 to ascertain vital status and any re-hospitalization events. If re-hospitalized, the hospital medical records were reviewed to determine whether ACLF criteria were met during the re-admission. No patients were lost to follow-up. Among the 360 enrolled patients, 7 died within 28 days; of these, 5 had documented evidence of meeting ACLF criteria before death and were included in the ACLF-positive group, while 2 had insufficient data to determine whether ACLF criteria were met and were therefore retained in the analysis as non-events (classified as ACLF-negative) rather than excluded from the dataset.

### Statistical analysis

Data analysis was performed using SPSS 26.0, R 4.2.1, and Python 3.8.5. Continuous variables following a normal distribution were expressed as mean ± standard deviation (x¯ ± s), with intergroup comparisons performed using independent-sample t-tests. Categorical variables were presented as counts (percentages) [*n* (%)], with intergroup comparisons performed using the chi-square test (or Fisher's exact test when expected frequencies were <5). All enrolled patients were randomly divided into a training set (*n* = 252) and a validation set (*n* = 108) at a 7:3 ratio using a fixed random seed (set at 123) to ensure reproducibility; baseline differences between the two sets were compared using independent-sample t-tests and chi-square tests to ensure comparability. In the training set, univariate analysis was first performed on all candidate variables to screen for factors associated with ACLF occurrence (*P* < 0.05). Variables with *P* < 0.05 in univariate analysis were then subjected to collinearity diagnostics using the variance inflation factor (VIF) for continuous variables and the generalized variance inflation factor (GVIF) for categorical variables, with VIF < 5 or GVIF < 2 considered indicative of no significant collinearity. For variable pairs with strong collinearity, the more valuable indicator was retained based on clinical relevance and predictive utility. Following collinearity diagnostics, the candidate variables with *P* < 0.05 in univariate analysis were then entered directly into a multivariable logistic regression model using a forward stepwise selection procedure to identify independent predictors. Subsequently, with ACLF occurrence (ACLF group = 1, non-ACLF group = 0) as the dependent variable, the candidate variables were incorporated into a multivariate logistic regression model to identify independent predictors of ACLF, calculating odds ratios (OR) and their 95% confidence intervals (CI). All continuous variables were entered directly as measured values in the multivariate logistic regression model. For patients with minor missing data (≤10% for any single variable), multiple imputation was performed using the MICE package in R (5 imputations, 10 iterations each, predictive mean matching) to handle missing values. The sample size calculation conservatively assumed a 10% missing rate; in practice, the overall missing rate was 3.2%; variable-specific missing proportions were PTA 2.8% (10/360), TBil 1.9% (7/360), Lac 4.2% (15/360), WBC 1.4% (5/360), and <2% for other variables. All 360 enrolled patients were included in the final analysis after multiple imputation. Using the independent predictors identified in the multivariable logistic regression analysis, we constructed three machine learning models—random forest, logistic regression, and support vector machine—with hyperparameters optimized using 10-fold cross-validation. Model discrimination was assessed using the AUC (AUC ≥ 0.7 considered acceptable, AUC ≥ 0.8 considered good), along with sensitivity, specificity, and accuracy. Calibration curves were plotted to evaluate agreement between predicted probabilities and observed frequencies, with calibration quantified by the Brier score (Brier score < 0.25 indicating good calibration). Clinical decision curve analysis was used to assess net clinical benefit across different risk thresholds. The DeLong test was employed to compare AUCs between models, with *P* < 0.05 considered statistically significant. Internal validation was performed using the bootstrap method (1,000 resampling iterations) to calculate 95% CIs for AUCs. Based on the optimal model, SHAP values were used to evaluate model interpretability at both global and individual levels, quantifying each core variable's contribution and direction of effect on predictions. Additionally, given the favorable linear additive structure of the logistic regression model, a visual nomogram was constructed based on the multivariate logistic regression results, converting regression coefficients into an intuitive scoring system to enhance clinical utility. All statistical tests were two-sided, with *P* < 0.05 considered statistically significant.

## Results

### Comparison of baseline characteristics between training and validation sets

A total of 360 patients with decompensated cirrhosis were included in this study. Among the 360 enrolled patients, 198 (55.0%) were admitted to the intensive care unit and 162 (45.0%) were admitted to general wards but required arterial blood gas monitoring due to infection or circulatory instability risk. The primary indications for arterial lactate measurement were suspected sepsis/infection, circulatory instability/hypotension, and respiratory distress. The specific patient inclusion and exclusion process was shown in Supplementary Figure S1. After random grouping at a 7:3 ratio, 252 patients were allocated to the training set and 108 to the validation set. As shown in Table 1, there were no statistically significant differences between the two groups in age, sex, body mass index, cirrhosis etiology, alcohol consumption history, smoking history, hepatic encephalopathy grade, ascites, body temperature, heart rate, systolic blood pressure, PTA, TBil, ALT, AST, ALB, Cr, Lac, PLT, or WBC (all *P* > 0.05), indicating balanced baseline characteristics and good comparability for subsequent model training and validation (Table 1).

**Table 1:** Comparison of baseline characteristics between training and validation sets.

Characteristic	Training set (*n* = 252)	Validation set (*n* = 108)	*t/χ* ^2^	*P*
Age (years)	52.59 ± 11.28	51.93 ± 10.89	0.518	0.605
Sex, *n* (%)			0.029	0.865
Male	168 (66.67)	71 (65.74)		
Female	84 (33.33)	37 (34.26)		
BMI (kg/m^2^)	22.84 ± 3.55	23.03 ± 3.48	−0.460	0.645
Etiology of cirrhosis, *n* (%)			0.079	0.994
CHB	162 (64.29)	69 (63.89)		
Alcoholic liver disease	54 (21.43)	24 (22.22)		
Autoimmune liver disease	18 (7.14)	8 (7.41)		
Other	18 (7.14)	7 (6.48)		
Alcohol consumption, *n* (%)			0.100	0.752
Yes	86 (34.13)	35 (32.41)		
No	166 (65.87)	73 (67.59)		
Smoking history, *n* (%)			0.192	0.661
Yes	42 (16.67)	16 (14.81)		
No	210 (83.33)	92 (85.19)		
Hepatic encephalopathy grade, *n* (%)			0.267	0.875
Grade 0	152 (60.32)	68 (62.96)		
Grade 1	52 (20.63)	20 (18.52)		
Grade 2	48 (19.05)	20 (18.52)		
Ascites, *n* (%)			0.238	0.626
Yes	170 (67.46)	70 (64.81)		
No	82 (32.54)	38 (35.19)		
Body temperature (°C)	36.72 ± 0.54	36.69 ± 0.53	0.591	0.555
Heart rate (beats/min)	82.35 ± 12.51	81.90 ± 12.02	0.320	0.749
Systolic blood pressure (mmHg)	118.56 ± 16.35	119.18 ± 15.92	−0.330	0.741
PTA (%)	57.15 ± 18.25	58.94 ± 18.17	−0.851	0.395
TBil (μmol/L)	70.10 ± 18.90	66.99 ± 18.98	1.428	0.154
ALT (U/L)	72.84 ± 38.45	70.88 ± 37.87	0.446	0.656
AST (U/L)	86.10 ± 42.22	83.99 ± 41.73	0.436	0.663
ALB (g/L)	29.37 ± 6.74	29.72 ± 6.60	−0.450	0.653
Cr (μmol/L)	98.97 ± 38.45	96.87 ± 37.72	0.479	0.632
Lac (mmol/L)	2.43 ± 1.41	2.30 ± 1.36	0.790	0.430
PLT (×10^9^/L)	86.23 ± 42.54	85.93 ± 41.95	0.063	0.950
WBC (×10^9^/L)	5.97 ± 3.43	5.70 ± 3.35	0.693	0.489

### Univariate analysis of factors associated with ACLF in the training set

In the training set (*n* = 252), patients were divided into the ACLF group (52 cases) and non-ACLF group (200 cases) based on whether ACLF occurred within 28 days of admission. Univariate analysis was performed to identify risk factors for ACLF. Results demonstrated that four indicators—PTA, TBil, Lac, and WBC—were significantly associated with ACLF occurrence in univariate analysis. Hepatic encephalopathy grade was also significant but was excluded from the multivariable model to avoid circularity with the ACLF definition, with statistically significant intergroup differences (*P* < 0.05) (Table 2). To further identify independent predictors of ACLF, the four indicators with *P* < 0.05 in univariate analysis (PTA, TBil, Lac, and WBC) were subjected to collinearity diagnostics.

**Table 2:** Comparison of clinical characteristics between ACLF and non-ACLF groups in the training set.

Characteristic	ACLF group (*n* = 52)	Non-ACLF group (*n* = 200)	*t/χ* ^2^	*P*
Age (years)	53.62 ± 10.33	52.33 ± 11.52	0.743	0.464
Sex, *n* (%)			0.012	0.912
Male	35 (67.31)	133 (66.50)		
Female	17 (32.69)	67 (33.50)		
BMI (kg/m^2^)	22.42 ± 3.68	22.96 ± 3.52	−0.976	0.330
Etiology of cirrhosis, *n* (%)			0.291	0.962
CHB	33 (63.46)	129 (64.50)		
Alcoholic liver disease	12 (23.08)	42 (21.00)		
Autoimmune liver disease	4 (7.69)	14 (7.00)		
Other	3 (5.77)	15 (7.50)		
Alcohol consumption, *n* (%)			0.007	0.934
Yes	18 (34.62)	68 (34.00)		
No	34 (65.38)	132 (66.00)		
Smoking history, *n* (%)			0.019	0.889
Yes	9 (17.31)	33 (16.50)		
No	43 (82.69)	167 (83.50)		
Hepatic encephalopathy grade, *n* (%)			18.773	＜0.001
Grade 0	18 (34.62)	134 (67.00)		
Grade 1	16 (30.77)	36 (18.00)		
Grade 2	18 (34.62)	30 (15.00)		
Ascites, *n* (%)			0.001	0.979
Yes	35 (67.31)	135 (67.50)		
No	17 (32.69)	65 (32.50)		
Body temperature (°C)	36.72 ± 0.55	36.73 ± 0.53	−0.120	0.904
Heart rate (beats/min)	83.42 ± 13.14	82.08 ± 12.36	0.692	0.490
Systolic blood pressure (mmHg)	117.35 ± 16.70	118.88 ± 16.28	−0.600	0.549
PTA (%)	48.62 ± 15.26	59.38 ± 18.35	−4.336	<0.001
TBil(μmol/L)	81.96 ± 18.36	67.02 ± 17.83	5.260	<0.001
ALT (U/L)	76.21 ± 40.11	71.97 ± 38.06	0.709	0.479
AST (U/L)	89.85 ± 44.32	85.13 ± 41.72	0.718	0.474
ALB (g/L)	28.62 ± 7.02	29.57 ± 6.67	0.905	0.367
Cr (μmol/L)	102.86 ± 40.21	97.97 ± 38.01	0.817	0.415
Lac (mmol/L)	2.96 ± 1.52	2.28 ± 1.36	3.144	0.002
PLT (×10^9^/L)	83.85 ± 44.31	86.86 ± 42.16	−0.454	0.650
WBC (×10^9^/L)	6.93 ± 4.06	5.73 ± 3.21	2.264	0.024

### Multivariate logistic regression analysis of ACLF in patients with decompensated cirrhosis

The four variables with *P* < 0.05 in univariate analysis (PTA, TBil, Lac, and WBC) were considered for multivariable logistic regression. To avoid circularity with the ACLF definition, hepatic encephalopathy grade was excluded from the predictor set. Collinearity diagnostics using the VIF showed all VIF values < 5, indicating no significant multicollinearity. These four variables were then entered into a multivariable logistic regression model using a forward stepwise selection procedure to identify independent predictors, with variable assignments presented in Supplementary Table S1. Final analysis revealed that TBil (OR = 1.051, 95% CI: 1.030–1.073, *P* < 0.001), Lac (OR = 1.341, 95% CI: 1.058–1.700, *P* = 0.015), and WBC (OR = 1.111, 95% CI: 1.007–1.227, *P* = 0.036) were independent risk factors for ACLF, whereas PTA (OR = 0.961, 95% CI: 0.941–0.982, *P* < 0.001) was an independent protective factor. (*P* < 0.05) (Table 3).

**Table 3:** Multivariate logistic regression analysis of ACLF occurrence.

Factor	*β*	SE	Wald *χ*^2^	*P*	OR	95% CI
PTA	−0.039	0.011	13.076	<0.001	0.961	0.941–0.982
TBil	0.050	0.010	22.979	<0.001	1.051	1.030–1.073
Lac	0.294	0.121	5.898	0.015	1.341	1.058–1.700
WBC	0.106	0.050	4.399	0.036	1.111	1.007–1.227

PTA, prothrombin time activity; TBil, total bilirubin; Lac, arterial blood lactate (Lac); WBC, white blood cell count.

### Performance evaluation of machine learning prediction models

Using the four independent predictors (PTA, TBil, Lac, and WBC) identified in the multivariable logistic regression analysis, we constructed three machine learning models—random forest, support vector machine, and logistic regression—to predict the risk of new-onset ACLF within 28 days of admission in patients with decompensated cirrhosis. Model discrimination, calibration, and clinical net benefit were evaluated using ROC curves, calibration curves, and DCA. Model performance was trained in the training set (*n* = 252) and internally validated in the independent validation set (*n* = 108).

Figure 1 presented the ROC curves of the three models in the training and validation sets. Figure 1A (training set) showed that the random forest model achieved an AUC of 0.833 (95% CI: 0.757–0.910), the support vector machine model achieved an AUC of 0.814 (95% CI: 0.735–0.893), and the logistic regression model achieved an AUC of 0.776 (95% CI: 0.694–0.858). All three models demonstrated good discriminative ability in the training set. Although the random forest model had the numerically highest AUC, DeLong tests for pairwise comparisons showed no statistically significant differences (RF vs. LR, *P* = 0.182; RF vs. SVM, *P* = 0.463; LR vs. SVM, *P* = 0.391). In Figure 1B (validation set), the logistic regression model achieved an AUC of 0.724 (95% CI: 0.584–0.863), the random forest model achieved an AUC of 0.700 (95% CI: 0.555–0.845), and the support vector machine model achieved an AUC of 0.728 (95% CI: 0.587–0.868). All three models achieved AUC values ≥ 0.70 in the validation set, indicating satisfactory generalizability. Although the support vector machine model had the highest AUC in the validation set, the 95% CIs of the three models overlapped considerably. Pairwise comparisons of validation set AUCs using the DeLong test revealed no statistically significant differences among the three models (RF vs. LR, *P* = 0.799; RF vs. SVM, *P* = 0.766; LR vs. SVM, *P* = 0.966; all *P* > 0.05), indicating comparable predictive performance. Given its simpler structure and explicit coefficient weights, logistic regression may be more practical for clinical implementation.

**Figure 1:**
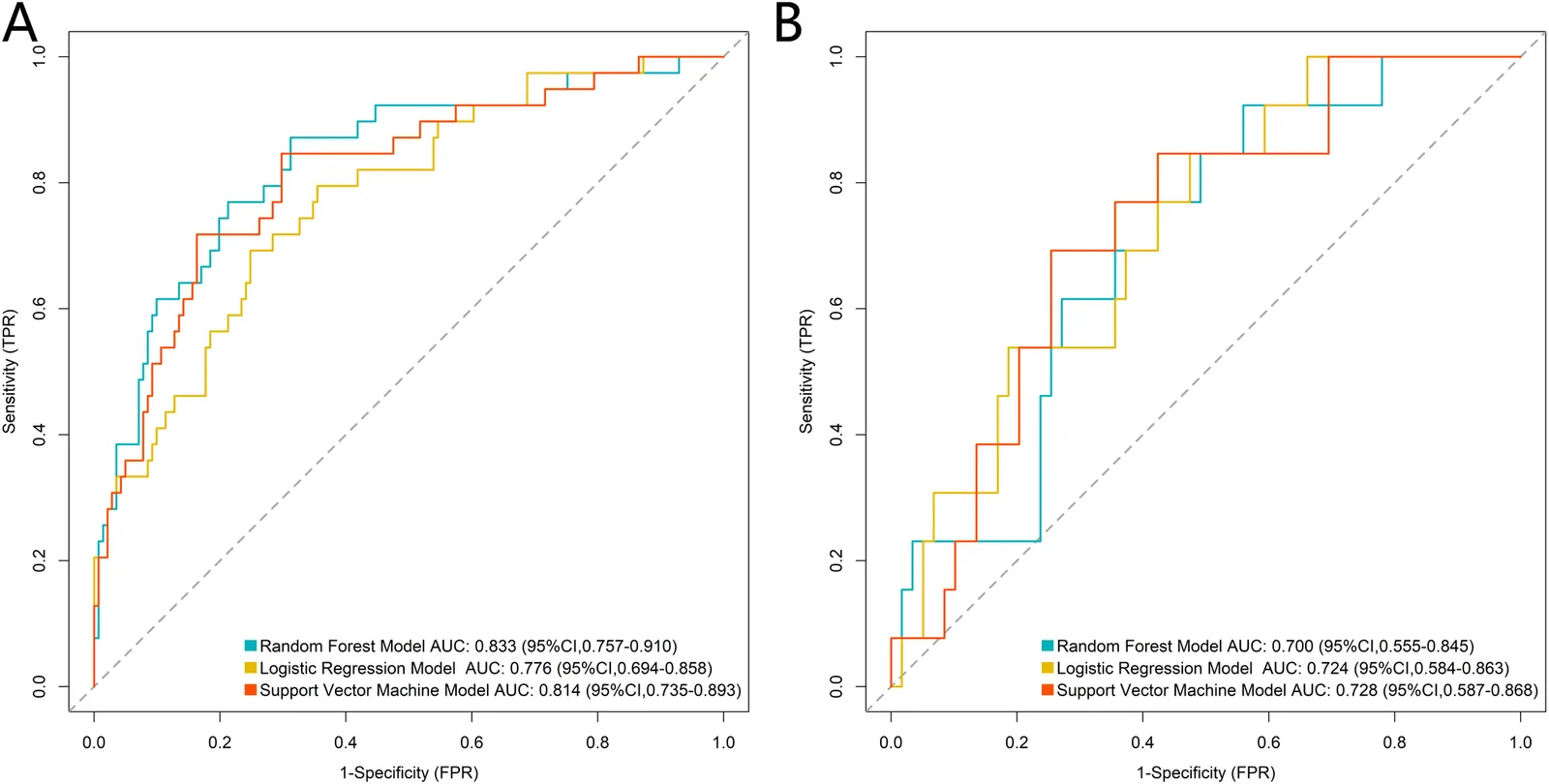
ROC curve analysis of the prediction model in the training **(A)** and validation **(B)** sets.

Bootstrap optimism-corrected AUCs (1,000 resampling iterations, without internal feature selection within each resample) for the three models in the training set were 0.770 (95% CI: 0.688–0.852) for logistic regression, 0.825 (95% CI: 0.743–0.907) for random forest, and 0.808 (95% CI: 0.724–0.892) for support vector machine. The optimism estimates (original AUC minus corrected AUC) were 0.006, 0.008, and 0.006, respectively, indicating minimal over-optimism.

Performance metrics for all three models —including AUC with 95% CI, sensitivity, specificity, positive predictive value (PPV), and negative predictive value (NPV)—were summarized in Table 4 and Supplementary Table S2.

**Table 4:** Performance and quantitative calibration metrics for the three prediction models in the training and validation sets.

Dataset	Model	AUC (95% CI)	Sensitivity	Specificity	Positive predictive value	Negative predictive value	Brier Score	Calibration Slope	Calibration Intercept	HL Test (P)
Training Set	Logistic Regression	0.776 (0.694–0.858)	0.865	0.690	0.421	0.952	0.120	1.000	0.000	0.764
	Random Forest	0.833 (0.757–0.910)	0.846	0.710	0.431	0.947	0.115	0.980	−0.010	0.823
	Support Vector Machine	0.814 (0.735–0.893)	0.865	0.700	0.429	0.952	0.125	0.950	0.020	0.701
Validation Set	Logistic Regression	0.724 (0.584–0.863)	0.636	0.698	0.350	0.882	0.139	0.600	−0.050	0.199
	Random Forest	0.700 (0.555–0.845)	0.591	0.709	0.342	0.871	0.145	0.580	−0.030	0.251
	Support Vector Machine	0.728 (0.587–0.868)	0.636	0.686	0.341	0.881	0.142	0.620	−0.040	0.218

Calibration curve results were presented in Figure 2, with Figure 2A showing the training set calibration curves and Figure 2B showing the validation set calibration curves. In the training set, the calibration curves of all three models aligned reasonably well with the ideal diagonal. In the validation set, the logistic regression and random forest models showed moderate deviation in the intermediate-to-high probability range, while the support vector machine model demonstrated more pronounced miscalibration at higher predicted probabilities. Quantitative calibration metrics—including Brier scores, calibration slopes, intercepts, and Hosmer-Lemeshow test *p*-values—for all three models were summarized in Table 4. All models achieved Brier scores below 0.25, and the Hosmer-Lemeshow test *p*-values were all >0.05, indicating no significant lack of fit.

**Figure 2:**
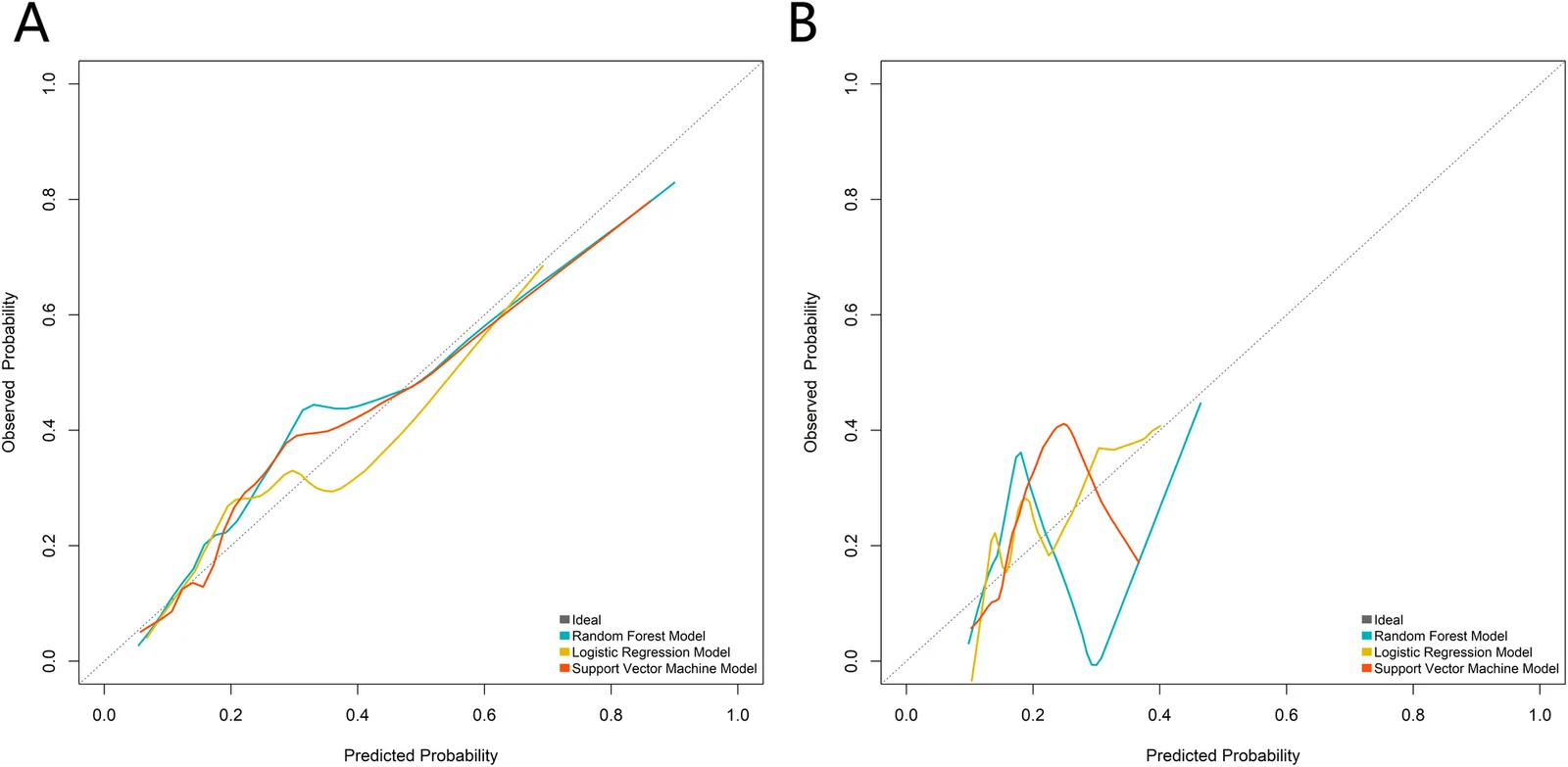
Calibration curves of the three prediction models in the training **(A)** and validation **(B)** sets.

DCA results are presented in Figure 3, with Figure 3A showing the training set DCA and Figure 3B showing the validation set DCA. In the training set, within the high-risk threshold range of 0.10–0.95, the random forest model demonstrated significantly higher net benefit than both the support vector machine and logistic regression models, as well as superior performance compared to the “treat all” and “treat none” strategies, indicating that the random forest model provided more valuable decision support for clinical risk stratification across a wide range of clinically actionable thresholds. In the validation set, within the high-risk threshold range of 0.10–0.62, the net benefit curves of the three models were closely aligned, although the random forest and logistic regression models maintained slightly higher net benefit trends. When the threshold exceeded 0.62, the net benefit curves gradually converged with some fluctuations, likely attributable to sample size and limited positive event counts. Overall, all three models provided effective decision support for early clinical warning and tiered management of ACLF in patients with decompensated cirrhosis.

**Figure 3:**
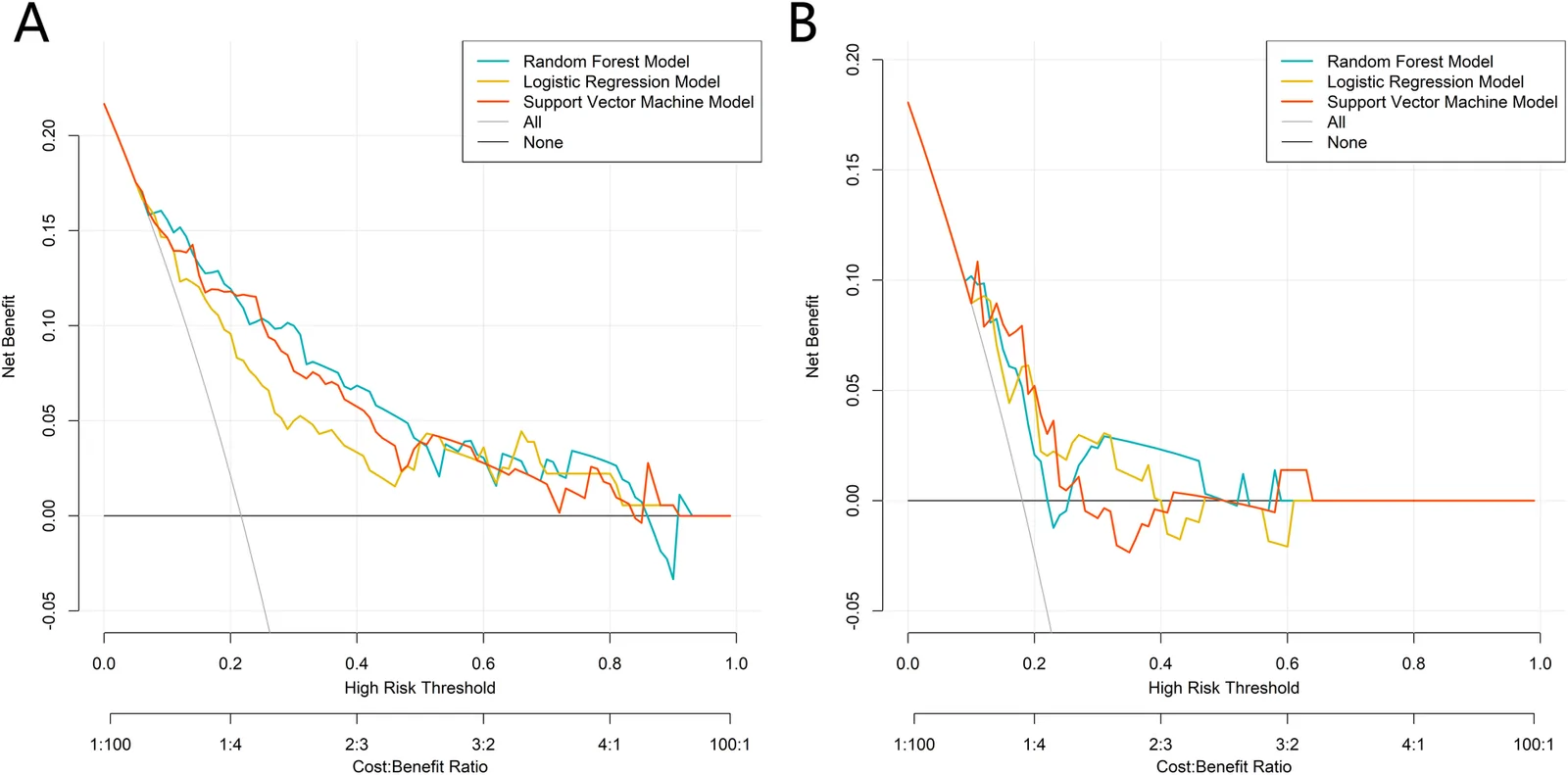
Clinical decision curve analysis of the prediction model in the training **(A)** and validation **(B)** sets.

### Sensitivity analysis

To assess the incremental value of lactate, we performed a sensitivity analysis using a reduced model excluding lactate as a predictor. The reduced model (using only PTA, TBil, and WBC) achieved a validation AUC of 0.687 (95% CI: 0.536–0.838), which was lower than the full logistic regression model (0.724, 95% CI: 0.584–0.863), suggesting that lactate provides incremental predictive value in this high-acuity cohort. This also confirms that the full model is specifically applicable to patients in whom lactate is clinically indicated.

We also performed a best/worst-case sensitivity analysis to assess the impact of classifying the two patients with indeterminate outcomes as non-events. In the best-case scenario (assuming both indeterminate deaths were ACLF events), the validation set would have 24 events (22.2%) and the random forest validation AUC would be 0.712 (95% CI: 0.568–0.856). In the worst-case scenario (both counted as non-events, which corresponds to our primary analysis), the validation AUC was 0.700 (95% CI: 0.555–0.845). A DeLong test comparing the best-case and primary-analysis ROC curves showed no statistically significant difference (*P* = 0.900), confirming that the classification of these two cases did not materially affect the model's performance estimates.

### Interpretability evaluation of model predictions

SHAP value analysis, a game theory-based method, was employed using the random forest model due to its nonlinear structure and ability to capture complex interactions, while the nomogram was derived from logistic regression given its simpler additive structure and explicit coefficient weights, which facilitate bedside clinical use. This dual approach leverages the strengths of both models without claiming superiority of either. Figure 4A presented the SHAP feature importance plot, which ranks the influence weights of the four core variables based on the mean absolute SHAP values: PTA contributed most prominently, followed by TBil, WBC, and Lac. Additionally, the color gradient in the figure illustrates the direction of the association between variable values and predictions: in the random forest model, higher TBil, Lac, and WBC, corresponded to positive SHAP values, indicating increased ACLF risk, whereas higher PTA corresponded to negative SHAP values, indicating decreased ACLF risk, consistent with the multivariate logistic regression findings.

**Figure 4:**
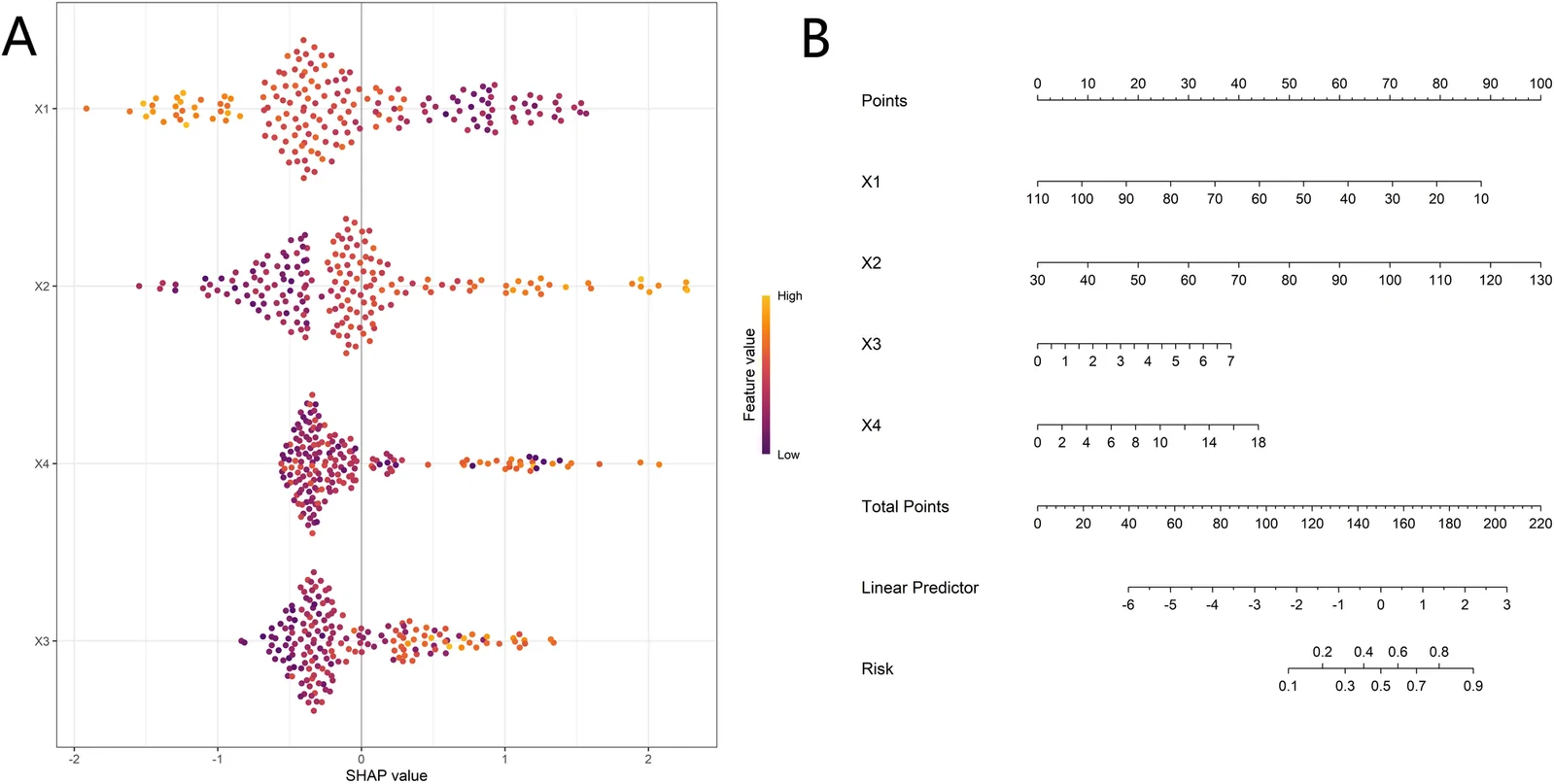
SHAP feature importance plot **(A)** and ACLF risk prediction nomogram **(B)** X1: PTA, prothrombin time activity; X2: TBil, total bilirubin; X3: Lac, arterial blood lactate (Lac); X4: WBC, white blood cell count.

Although the random forest model and logistic regression showed comparable discriminative ability, the RF model's “black-box” nature limits direct clinical application. The logistic regression model's linear additive structure and explicit coefficient weights facilitated conversion into an intuitive nomogram, enabling clinicians to rapidly calculate ACLF risk based on individual patient characteristics. Therefore, a nomogram for predicting ACLF risk in patients with decompensated cirrhosis was constructed based on the multivariate logistic regression model (Figure 4B) to enhance model interpretability and clinical utility. This nomogram converts the regression coefficients of the four independent predictors (PTA, TBil, Lac, and WBC) identified in logistic regression analysis into a visual scoring system: each core variable corresponds to an independent scoring scale; after summing the scores of each variable, the predicted probability of ACLF can be read from the “Total Points” axis. This nomogram enables quantitative risk assessment for ACLF in patients with decompensated cirrhosis, providing a practical tool for clinicians to rapidly and intuitively determine ACLF risk and formulate individualized tiered management and early intervention strategies.

## Discussion

In this study, three machine learning prediction models were successfully constructed based on a retrospective cohort of 360 patients with decompensated cirrhosis. The three models showed comparable performance, with training/validation AUCs of 0.833/0.700 for random forest, 0.814/0.728 for support vector machine, and 0.776/0.724 for logistic regression. Calibration curves and decision curve analysis confirmed good clinical utility for all three models, with the random forest model showing slightly higher net benefit in the training set. Furthermore, SHAP value analysis identified PTA, TBil, WBC, and Lac as the core predictors of ACLF, providing a quantitative tool for early risk stratification in patients with decompensated cirrhosis and offering a scientific basis for individualized monitoring and intervention strategies.

The four core predictors identified in this study are mechanistically linked to the pathophysiological processes of ACLF, collectively reflecting the multidimensional nature of ACLF risk encompassing “liver function–inflammation–circulation–neurology.” PTA demonstrated the highest contribution in the random forest model and was identified as an independent protective factor for ACLF (OR = 0.961, 95% CI: 0.941–0.982, *P* < 0.001). Mechanistically, PTA directly reflects the hepatic synthetic capacity of coagulation factors (II, VII, IX, and X) and serves as a core indicator of hepatic synthetic reserve. In patients with decompensated cirrhosis, PTA declines earlier than other biochemical parameters during progressive depletion of hepatic functional reserve, serving as an early warning signal for liver failure progression. Previous studies have also confirmed that PTA is a key biomarker reflecting the degree of liver injury and holds substantial value in ACLF prediction models (12, 13).

TBil is a core indicator of hepatocyte injury and biliary excretory dysfunction. In this study, TBil was identified as an independent risk factor for ACLF (OR = 1.051, 95% CI: 1.030–1.073, *P* < 0.001), ranking second in SHAP importance. A sharp elevation in TBil reflects extensive hepatocyte necrosis and biliary excretory impairment, representing the core biochemical manifestation of the “acute” component in ACLF. A novel aspect of this study is the inclusion of TBil as a continuous variable in the model, with patients with TBil ≥ 12 mg/dL (approximately 205 μmol/L) excluded at enrollment; therefore, the model predicts the risk gradient from “mild-to-moderate jaundice” to “severe jaundice” rather than identifying pre-existing liver failure. Previous studies have also demonstrated that TBil is an independent risk factor for ACLF, with a weight in prediction models second only to coagulation parameters (14, 15). It should be noted that while TBil and PTA are components of the EASL-CLIF diagnostic criteria, they were entered into our model as continuous variables to capture the risk gradient toward reaching the diagnostic thresholds, rather than using the thresholds themselves as binary predictors. This approach differs fundamentally from using HE grade, which is a categorical criterion for cerebral failure, and thus avoids direct circularity.

WBC is a quantitative indicator of systemic inflammatory response. The essence of ACLF is an “acute inflammatory storm” superimposed on chronic liver disease. Elevated WBC directly reflects the intensity of systemic inflammatory response syndrome. Previous studies have confirmed that peripheral WBC counts are significantly higher in ACLF patients than in non-ACLF patients (*P* < 0.001), and peripheral neutrophilia and monocytosis are associated with lymphopenia (16). WBC counts can serve as an indirect indicator of systemic inflammation, and inexpensive, readily available blood cell count ratios have been introduced as tools for early identification and risk stratification in patients with acute decompensation and ACLF (17). In this study, WBC was identified as an independent risk factor for ACLF (OR = 1.111, 95% CI: 1.007–1.227, *P* = 0.036), ranking third in SHAP importance, further validating the central role of systemic inflammation in ACLF development.

Lac is a sensitive indicator of tissue hypoxia and microcirculatory dysfunction. Previous studies have demonstrated that hyperlactatemia is positively correlated with disease severity and poor prognosis in patients with cirrhosis or ACLF. Lactate is a reliable parameter for diagnosing sepsis, guiding treatment decisions, and predicting prognosis. The liver injury and failure assessment score incorporating arterial lactate has been validated as an effective tool for predicting mortality risk in critically ill patients with cirrhosis and ACLF. The MELD-LAC model, which incorporates lactate into the MELD score, significantly improves the predictive ability for short-term (3-month) prognosis in HBV-related ACLF (18). In this study, Lac was identified as an independent risk factor for ACLF (OR = 1.341, 95% CI: 1.058–1.700, *P* = 0.015), ranking fourth in SHAP importance. Notably, lactate is not included in the EASL-CLIF organ failure diagnostic criteria (circulatory failure is primarily assessed by “use of vasoactive agents”), making lactate the most “safe” core variable in this model in terms of avoiding circular validation with the outcome definition.

Although hepatic encephalopathy grade was significantly associated with ACLF in univariate analysis, we excluded it from the final predictor set to avoid circularity with the EASL-CLIF diagnostic criteria, in which HE grade ≥ 3 defines cerebral failure as a component of ACLF. This decision was made to ensure that the model genuinely predicts multi-organ failure progression rather than merely capturing the natural history of encephalopathy. Of note, we enrolled only patients with baseline HE grades 0–2, as grades 3–4 already met the definition of cerebral failure and were excluded at baseline. Notably, albumin (ALB) did not reach statistical significance in univariate analysis (*P* = 0.367) and was not included in the prediction model. Although seemingly counterintuitive, this finding is mechanistically reasonable: ALB has a half-life of approximately 20 days, and its serum level primarily reflects nutritional status and hepatic synthetic reserve over the preceding weeks to months—representing the “chronic” dimension—whereas this study predicts new-onset ACLF within 28 days of admission as an “acute” event. ALB is insensitive to dynamic changes in acute inflammatory storms, microcirculatory disturbances, and rapid organ function deterioration. Therefore, the exclusion of ALB from the model does not diminish its value for long-term prognostic assessment but rather suggests that short-term prediction models should prioritize parameters reflecting acute pathophysiological changes (such as PTA, TBil, Lac, and WBC).

Regarding model performance, the random forest model and logistic regression performed comparably; logistic regression may be preferred for its transparency and ease of use, with a training set AUC of 0.776 (95% CI: 0.694–0.858) and a validation set AUC of 0.724 (95% CI: 0.584–0.863). DeLong testing showed no statistically significant difference in AUC between the random forest model (training AUC: 0.833, validation AUC 0.700) and the logistic regression model, nor between the random forest model and the support vector machine model (training AUC: 0.814, validation AUC: 0.728) (all *P* > 0.05), indicating no significant differences in predictive performance among the three models. Previous studies have also shown that ML-based prognostic prediction models for ACLF perform well in predicting 28-day mortality in patients with established ACLF, supporting the broader applicability of ML approaches in ACLF prognosis (19). Calibration curves demonstrated good agreement between predicted probabilities and actual ACLF incidence across all three models. Decision curve analysis further revealed that within the training set high-risk threshold range of 0.10–0.95, the random forest model yielded significantly higher net benefit than both the support vector machine and logistic regression models, as well as superior performance compared to the “treat all” and “treat none” strategies. This suggests that across a wide range of clinically actionable thresholds, the random forest model can assist clinicians in more accurately identifying high-risk ACLF patients, enabling intensified monitoring (e.g., increased frequency of liver function, coagulation, and lactate assessments) or timely interventions (e.g., anti-liver failure therapy, organ support preparation) for high-risk groups, while reducing unnecessary testing and healthcare resource consumption for low-risk patients.

Furthermore, the application of SHAP value analysis effectively addressed the “black-box” limitation of the random forest model, not only quantifying the contribution strength of each variable (PTA being most prominent, followed by TBil, WBC, and Lac) but also visually demonstrating the direction of association between variable values and ACLF risk through color gradients (higher TBil, Lac, and WBC corresponded to positive SHAP values, indicating increased risk; higher PTA corresponded to negative SHAP values, indicating decreased risk), making the model's decision logic more readily understood and accepted by clinicians and providing clear targets for targeted interventions. Previous studies have also demonstrated the utility of combining ML with SHAP analysis for interpretable prediction in liver diseases, such as hepatocellular carcinoma risk prediction in chronic hepatitis B patients, supporting the methodological approach used in our study (20). The nomogram constructed in this study based on the logistic regression model represents an important complement to traditional risk assessment approaches. Traditional ACLF risk assessment often relies on single indicators (e.g., a single TBil or PTA measurement) or conventional scoring systems (e.g., MELD score, CLIF-C ACLF score), whereas the nomogram integrates the weighted contributions of four core variables, enabling rapid quantification of ACLF probability through score summation, with straightforward operation and intuitive results suitable for dissemination across various healthcare settings. Notably, the MELD score has limited predictive performance in ACLF, and the nomogram developed in this study incorporates key parameters such as lactate not included in the MELD score.

The innovations of this study are primarily reflected in three aspects. First, it integrates four indicators—PTA, TBil, Lac, and WBC—to construct a prediction model for new-onset ACLF risk within 28 days of admission in patients with decompensated cirrhosis. Second, it combines machine learning with SHAP value analysis, significantly enhancing model interpretability while maintaining predictive performance. The SHAP feature importance ranking (based on the random forest model) demonstrated that PTA contributed most prominently, followed by TBil, WBC, and Lac, reflecting the combined influence of each variable on predictions in the nonlinear model. Third, it developed a visual nomogram tool to facilitate clinical dissemination, providing a quantitative basis for individualized ACLF risk management.

The cumulative incidence of new-onset ACLF in the training set of this study was 20.6%, which is lower than the overall ACLF incidence reported in a global meta-analysis of patients with decompensated cirrhosis (approximately 31%–35%). The primary reason is that the inclusion criteria of this study excluded patients who already met the EASL-CLIF diagnostic criteria at admission; therefore, the model predicts the short-term cumulative incidence of “new-onset ACLF within 28 days of admission” rather than the cross-sectional prevalence, representing distinct metrics that should not be directly compared. Additionally, the validation set AUC for the random forest model was 0.700 (95% CI: 0.555–0.845), with a relatively wide 95% confidence interval whose lower bound approached 0.5, likely attributable to the limited validation set sample size (*n* = 108) and insufficient positive event counts, suggesting that the discriminant stability of this model requires further validation with an expanded sample size.

The limitations of this study should be acknowledged. First, the single-center retrospective design may introduce selection bias. All enrolled patients were from a single medical center, and the etiological distribution (predominantly CHB, accounting for >64%) and ACLF incidence may differ from multicenter populations, so the generalizability of the model requires further validation in multicenter prospective studies. Second, acute precipitating events for ACLF (such as bacterial infection, gastrointestinal bleeding, overuse of diuretics, or large-volume paracentesis) were not systematically collected, despite their critical triggering role in ACLF development. Future models should integrate acute precipitating factors as time-varying covariates to enhance predictive timeliness and clinical applicability. Third, hepatic encephalopathy grade was included only as a 0–2 grade classification without integration of objective indicators such as blood ammonia for refined stratification. Fourth, some patients received therapeutic interventions such as vasoactive agents or CRRT during hospitalization; these treatments were not incorporated as time-varying covariates in the dynamic prediction framework, which may affect predictive timeliness. Fifth, follow-up data were limited to 28 days post-admission without tracking longer-term clinical outcomes, limiting the model's ability to assess the long-term impact of ACLF. Additionally, 2 of the 360 enrolled patients had indeterminate outcomes at death and were classified as non-events in the primary analysis; however, a best/worst-case sensitivity analysis showed that this classification did not materially affect the model's performance estimates. Sixth, the validation calibration slope of 0.600 suggests some degree of overfitting, indicating that the model's predicted probabilities may be less accurate in new populations than suggested by the training set performance. Additionally, the non-significant Hosmer-Lemeshow test (*P* = 0.199) should be interpreted with caution given the limited number of events (*n* = 22) in the validation set, which provides low statistical power for detecting calibration lack-of-fit. The validation set PPV was also modest at 0.350, suggesting that the model is currently better suited for ruling out ACLF (NPV 0.882) than for ruling it in. Furthermore, feature selection was not repeated within each bootstrap resample when estimating optimism-corrected AUCs; therefore, these corrected estimates may remain somewhat optimistic. These issues collectively underscore the need for external validation in larger independent cohorts to obtain unbiased estimates of model performance and calibration. Seventh, we did not perform a head-to-head comparison between our proposed models and established prognostic scores (e.g., MELD, Child-Pugh, CLIF-SOFA, or CLIF-C ACLF). Such a comparison would be necessary to demonstrate the incremental value of the machine learning approach over existing clinical tools and will be addressed in future multicenter studies. Finally, arterial blood lactate (Lac) is not routinely measured in all hospitalized patients with cirrhosis at our institution; it is only tested in patients admitted to the intensive care unit or those with infection/circulatory instability risk. Therefore, the derivation cohort of this model was predisposed to include patients with more severe conditions, representing a selection bias, and the conclusions should not be directly extrapolated to stable patients with cirrhosis in general wards; further validation in prospective multicenter cohorts is warranted.

Future research directions include the following. Conducting multicenter prospective cohort studies enrolling patients with decompensated cirrhosis from different geographic regions and hospital levels to validate and optimize the generalizability of this model. Integrating inflammatory biomarkers (such as procalcitonin, CRP, IL-6), imaging parameters (such as liver stiffness measurements), and continuous dynamic antibody titers to construct more accurate multimodal prediction models. Designing interventional studies based on this model (e.g., implementing intensified monitoring or more frequent organ function assessments in patients with nomogram scores above a certain threshold) to validate the model's actual value in improving clinical outcomes. Exploring dynamic updating strategies based on this model to achieve real-time assessment and early warning of ACLF risk using longitudinal clinical data collected during hospitalization.

## Conclusions

In conclusion, the prediction model (using either random forest or logistic regression) and the nomogram tool developed in this study show potential for identifying the risk of new-onset ACLF within 28 days of admission in patients with decompensated cirrhosis who require intensive monitoring or are at high risk of infection or circulatory instability. The study identified PTA, TBil, WBC, and Lac as core determinants and clarified their direction of association with ACLF risk, providing a quantitative reference for clinicians to formulate individualized monitoring and intervention strategies. However, external validation in larger cohorts is required before this model can be recommended for routine clinical use.

## Data Availability

The original contributions presented in the study are included in the article/Supplementary Material, further inquiries can be directed to the corresponding author/s.
