# Supplementary material for: Machine learning model for ACLF risk in decompensated cirrhosis
Source: Front Med (Lausanne). 2026 Oct 7;13:1948234. doi: 10.3389/fmed.2026.1948234 (PMC13683224; doi:10.3389/fmed.2026.1948234)
Supplement: Supplementary file 2 [file Datasheet2.docx]

**Supplemental Table 1.** Variable assignment

| Variable | Meaning | Assignment |
| --- | --- | --- |
| X1 | PTA | Continuous variable |
| X2 | TBil | Continuous variable |
| X3 | Lac | Continuous variable |
| X4 | WBC | Continuous variable |
| Y | ACLF occurrence | ACLF group = 1, non-ACLF group = 0 |

**Supplementary Table 2.** Confusion matrices for the three prediction models in the training and validation sets

| Dataset | Model | TP | FN | TN | FP |
| --- | --- | --- | --- | --- | --- |
| Training Set | Logistic Regression | 45 | 7 | 138 | 62 |
| (n=252, events=52) | Random Forest | 44 | 8 | 142 | 58 |
|  | Support Vector Machine | 45 | 7 | 140 | 60 |
| Validation Set | Logistic Regression | 14 | 8 | 60 | 26 |
| (n=108, events=22) | Random Forest | 13 | 9 | 61 | 25 |
|  | Support Vector Machine | 14 | 8 | 59 | 27 |

TP: true positives; FN: false negatives; TN: true negatives; FP: false positives. Metrics are reported for the re-trained four-variable model.
