# Supplementary material for: Machine learning model for ACLF risk in decompensated cirrhosis
Source: Front Med (Lausanne). 2026 Oct 7;13:1948234. doi: 10.3389/fmed.2026.1948234 (PMC13683224; doi:10.3389/fmed.2026.1948234)
Supplement: Supplementary file 3 [file Datasheet1.docx]

**Supplementary Figure 1** Flow chart of patient screening
